# Supplementary material for: A Quality Improvement Initiative to Reduce Postoperative Delirium among Cardiac Surgery Patients
Source: Geriatrics (Basel). 2021 Nov 16;6(4):111. doi: 10.3390/geriatrics6040111 (PMC8628740; doi:10.3390/geriatrics6040111)
Supplement: Supplementary file 1 [file geriatrics-06-00111-s001.zip › geriatrics-1440779-supplementary.pdf]

Table S1. Delirium Quality improvement.

Timeline	Event	Location	Surgical phase	Actions	Purpose	Comments
2009-2010	Delirium Screening-research project	ICCS	Preoperative	CAM- education and information	Staff education	Delirium Assessment
		CSIU	Preoperative	CAM- education and information		
2010	Delirium screening	ICCS	Preoperative	CAM-ICU (2/day)	Delirium Screening	Delirium assessment
		CSIU	Preoperative	CAM		
	Delirium Decision tree	CSIU	Preoperative	Risk factors + CAM + RASS	A combined evaluation of preexisting risk factors, confusion and agitation	Risk factor and Delirium Assessment
2012	WRHA surgical delirium program	CSIU	Preoperative	CAM- education and information	Staff education	Delirium Assessment
	Fraillty evaluation	CSIU	Preoperative	CFS	Evaluate frailty as a risk factor of delirium	Risk factor evaluation
		Cardiac surgeon	Preoperative			
	Cardiac science transdisciplinary delirium working group	Cardiac science program	-	A transdisciplinary team to plan and guide delirium program implementation in various cardiac surgery units	Delirium project planning and implementation, Delirium management – guideline development	leadership
2013	Preoperative assessment package	CPAC	Preoperative	MoCA, PHQ-9, CFS	Delirium, Depression, Frailty	Risk factor and Delirium assessment
	WRHA delirium brochure	Preoperative cardiac surgery package	Preoperative	Preoperative mailing package		Preoperative delirium education and assessment
	"Getting to know you" form					
	'Primary care physician' - letter		Preoperative	To inform FP of the patients high risk of depression		Delirium prevention
2013-2014	Cardiac science-delirium guideline development	Cardiac science program	The goal was to adapt WRHA delirium program with specific additions related to cardiac surgery population			
2014	Cardiac science delirium scoring system	CPAC	Preoperative	The pre assessment screening test	Baseline risk factor assessment, MoCA, PHQ-9, AUDIT	Risk factor and delirium assessment
	Delirium Screening	ICCS	Postoperative	CAM-4 hourly		Delirium screening

	Pain assessment	ICCS	Postoperative	CPOT	Asses level of pain	Risk of delirium assessment
**2015**	Cardiac science family brochure				Family mental health	Family support
	Delirium pre op assessment package	CSIU, Cardiology In-patient, CPAC, CRIU	Preoperative	Baseline risk factor, MoCA, PHQ-9, AUDIT		Delirium assessment
	Delirium order set	ICCS	Postoperative	Medication orders, General orders<br>Adjunct therapy, Additional consideration	Support clinical judgment and clinical practice	Delirium prevention and management
	'primary care physician' - letter		Postoperative		To inform delirium occurrence	Delirium management
	Cardiac surgical time out process	Operative	Operative	Delirium Score		Delirium prevention
**2016**	Early mobility	ICCS	Postoperative	Early mobility		Delirium prevention
	Nursing education delirium module	Cardiology inpatient unit, ICCS, CPAC, CSIU				Staff education

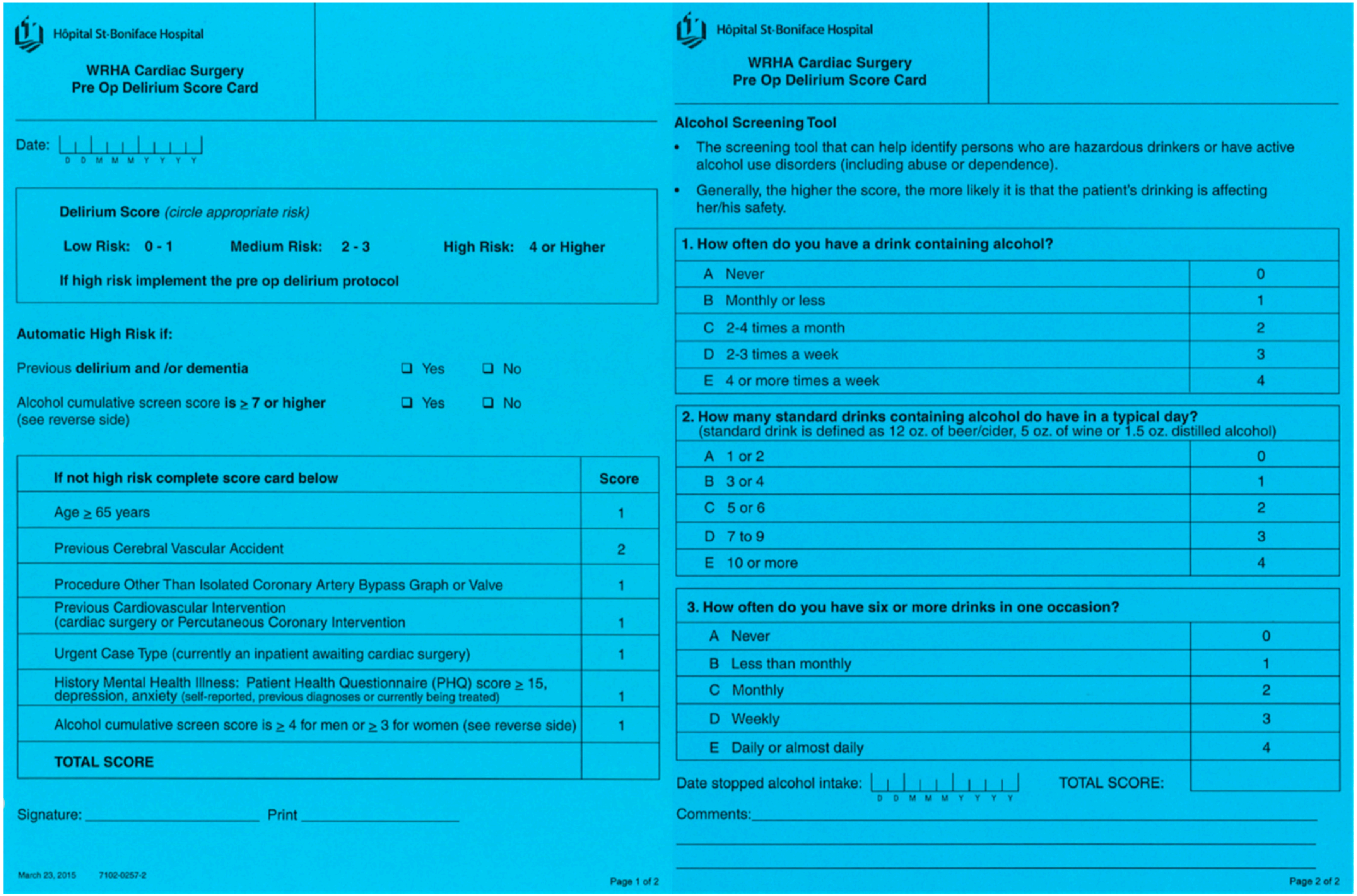

Hôpital St-Boniface Hospital

WRHA Cardiac Surgery
Pre Op Delirium Score Card

Date: D D M M M Y Y Y Y

Delirium Score *(circle appropriate risk)*

Low Risk: 0 - 1 **Medium Risk: 2 - 3** **High Risk: 4 or Higher**

If high risk implement the pre op delirium protocol

Automatic High Risk if:

Previous **delirium and /or dementia** ❑ Yes ❑ No

Alcohol cumulative screen score **is ≥ 7 or higher** ❑ Yes ❑ No
(see reverse side)

**If not high risk complete score card below**	**Score**
Age ≥ 65 years	1
Previous Cerebral Vascular Accident	2
Procedure Other Than Isolated Coronary Artery Bypass Graph or Valve	1
Previous Cardiovascular Intervention (cardiac surgery or Percutaneous Coronary Intervention	1
Urgent Case Type (currently an inpatient awaiting cardiac surgery)	1
History Mental Health Illness: Patient Health Questionnaire (PHQ) score ≥ 15, depression, anxiety (self-reported, previous diagnoses or currently being treated)	1
Alcohol cumulative screen score is ≥ 4 for men or ≥ 3 for women (see reverse side)	1
**TOTAL SCORE**	

Signature: ____________________ Print ____________________

March 23, 2015 7102-0257-2 Page 1 of 2

Hôpital St-Boniface Hospital

WRHA Cardiac Surgery
Pre Op Delirium Score Card

Alcohol Screening Tool

- The screening tool that can help identify persons who are hazardous drinkers or have active alcohol use disorders (including abuse or dependence).
- Generally, the higher the score, the more likely it is that the patient's drinking is affecting her/his safety.

**1. How often do you have a drink containing alcohol?**	
A Never	0
B Monthly or less	1
C 2-4 times a month	2
D 2-3 times a week	3
E 4 or more times a week	4

**2. How many standard drinks containing alcohol do have in a typical day?** (standard drink is defined as 12 oz. of beer/cider, 5 oz. of wine or 1.5 oz. distilled alcohol)	
A 1 or 2	0
B 3 or 4	1
C 5 or 6	2
D 7 to 9	3
E 10 or more	4

**3. How often do you have six or more drinks in one occasion?**	
A Never	0
B Less than monthly	1
C Monthly	2
D Weekly	3
E Daily or almost daily	4

Date stopped alcohol intake: D D M M M Y Y Y Y TOTAL SCORE:

Comments: ____________________

Page 2 of 2

Figure S1. Delirium score card.
